# The burden of COVID-19 in French Guiana: Vaccine-averted deaths, hospitalizations and costs

**DOI:** 10.1016/j.jvacx.2023.100271

**Published:** 2023-02-11

**Authors:** Mathieu Nacher, Nicolas Vignier, Cyril Rousseau, Antoine Adenis, Maylis Douine, Célia Basurko, Bertrand de Toffol, Narcisse Elenga, Hatem Kallel, Jean Pujot, Magaly Zappa, Magalie Demar, Félix Djossou, Pierre Couppié, Loïc Epelboin

**Affiliations:** a CIC INSERM 1424, Centre Hospitalier de Cayenne, 97300 French Guiana; b Département Formation Recherche, Université de Guyane, French Guiana; c Santé Publique France, French Guiana; d Service de Neurologie, Centre Hospitalier de Cayenne, 97300 French Guiana; e Service de Pédiatrie, Centre Hospitalier de Cayenne, 97300 French Guiana; f Service de Réanimation, Centre Hospitalier de Cayenne, 97300 French Guiana; g Service de, Centre Hos Urgences, Centre Hospitalier de Cayenne, 97300 French Guiana; h Service de Radiologie, Centre Hospitalier de Cayenne, 97300 French Guiana; i Laboratoire polyvalent, Centre Hospitalier de Cayenne, 97300 French Guiana; j Unité Mixte de RechercheTropical Biome and Immunopathology, Université de Guyane, French Guiana; k Service des Maladies Infectieuses et Tropicales, Centre Hospitalier de Cayenne, 97300 French Guiana; l Service de Dermatologie, Centre Hospitalier de Cayenne, 97300 French Guiana

**Keywords:** COVID-19, Vaccination, Vaccine reluctance, Number needed to vaccinate, Mortality, Cost, Diffusion of Innovations

## Abstract

**Objectives:**

French Guiana, the least-vaccinated French territory, also has the lowest COVID-19 vaccination coverage in Latin America. We aimed to estimate how many deaths, hospitalizations and costs the vaccines had and could have avoided.

**Methods:**

We calculated the Number Needed to Vaccinate to prevent one death per year, 1 standard hospitalization, 1 Intensive Care Unit admission given the mean incidence numbers of the past 6 months, and divided the number of persons vaccinated to estimate how many deaths and hospitalizations had been avoided in French Guiana at that time.

**Results:**

The crude number needed to vaccinate to prevent one death per year, the crude number needed to vaccinate to prevent one hospitalization per 6 months were computed Based on our observed incidence and ICU admission rate, the crude number needed to vaccinate to prevent one ICU admission per 6 months.

After 6 months with an incidence exceeding 400 per million inhabitants, and 148 observed deaths, we estimate that vaccination avoided 46 deaths (IC95%=43.5–48.7). If the number of vaccinated persons had reached the same proportion as mainland France, 141 deaths per year could have been prevented (IC95%=131.9–147.6).

With 2085 hospitalization and 370 ICU admissions during the same period, we estimate that the current albeit low vaccination rate avoided 300 hospital (IC95%=280–313) and 77 (IC95%=72–81) ICU admissions. With the same vaccination rates as mainland France, we estimate that 900 hospitalizations and 231 ICU admissions would have been avoided.

Similarly, there would have been 139 ICU admission (instead of 370).

**Conclusions:**

In sparsely populated French Guiana these numbers are quite substantial and framing the vaccine benefits and wasted opportunities using such concrete numbers may help convincing undecided persons to get vaccinated.

## Highlights

• French Guiana, the least-vaccinated French territory, also has the lowest COVID-19 vaccination coverage in Latin America.
• After 6 months with an incidence exceeding 400 per million inhabitants, and 148 observed deaths, we estimate that vaccination avoided 300 hospital and 77 ICU admissions, and 46 deaths.
• If the number of vaccinated persons had reached the same proportion as mainland France, 900 hospitalizations and 231 ICU admissions and 141 deaths per year could have been prevented during that period.
• The vaccine gap relative to France led to an excess of 43% of hospitalizations 62.4% of ICU admissions, and 64% of deaths.

## 1 Introduction

French Guiana, a French territory located between Brazil and Suriname, has the highest GDP per capita in Latin America. It is a young (median age 23 years) sparsely populated (circa 300 000 inhabitants) but vast territory (the size of Portugal). It is culturally very diverse with Mainland French, Creoles from French Guiana and the French Caribbean, Maroons, Amerindians, Hmong, Chinese, Haitians, Brazilians, Surinamese, Guyanese, Dominicans, Peruvians… among others. Apart from COVID19, general vaccine coverage in French Guiana is lower than in France, mostly in relation with social and territorial inequalities in health.[1] Despite being lower or slower than in mainland France, vaccination in French Guiana reached a substantial proportion of the population for various types of vaccines (recombinant, inactivated, live vaccines). As other territories in the Amazon region, French Guiana has been severely affected by COVID-19[2], [3]. Its particular status –French and in South America— had concrete epidemiological consequences as new waves could arrive from France (through the airport) or from Brazil (across the border), with some seasonality since when most of Brazil was in winter, France was in Summer, and vice versa. Since January 2021, the Pfizer-BioNTech COVID-19 vaccine has been rapidly rolled out free of charge with much broader indications than in mainland France, and most neighboring countries, particularly Brazil and Suriname. Despite this relatively early start and great efforts from the local and national health authorities to communicate and reach out to the most socially isolated populations, as of April 22, only 29.8 % of the population had completed vaccination. In contrast, in mainland France 79.3 % of the total population had completed their COVID-19 vaccination (not including booster doses).[4] Furthermore, while French Guiana was the first South American territory to have access to the vaccine it is now the last one in terms of vaccine coverage, behind Venezuela and Bolivia 2 countries with the lowest GDP per capita. In this context, back to back third and fourth waves of COVID-19 stretched from April 2021 to October 2021 (Gamma variant until mid-April and then on Delta variant until present) profoundly straining French Guiana’s health system with hospital beds and ICU beds fully occupied by COVID-19 patients requiring reinforcements by health professionals from mainland France[5]and medical evacuations to ICUs in Martinique or Mainland France.[6]The Health PASS control that was implemented in mainland France and vaccine mandates for health professionals –a substantial proportion of whom refuse to get vaccinated[7]—were postponed in French Guiana and the French Antilles. In French Guiana, a cross-sectional survey between January and March 2021 hence revealed that 24.3 % of health did not want to get vaccinated and 11.2 % were unsure.[7] Younger health professionals, those who were not worried by COVID-19, non– medical professions, those who did not trust authorities or the pharmaceutical industry, those who feared side effects, and those who were born in French Guiana were more likely to have negative attitudes towards COVID-19 vaccination. [7], [8].

In this context of distrust fueled by social media, an aggressive antivaccine movement tried to recruit the population against COVID-19 vaccination, frequently resorting to intimidation, threats and sometimes violence against health professionals and journalists.[9], [10].

Despite the profound health and economic crisis, many of the most prominent political figures have thus taken positions that avoided upsetting antivaccine advocates and compromising local political coalitions. In late September 2021, however, a number of opinion leaders and some politicians started publicly taking positions where they encourage the local population to get vaccinated. [11] Vaccine hesitancy is defined by the World Health Organization (WHO) as a behavior, influenced by a number of factors that include issues of confidence, complacency (there is no need for a vaccine), and convenience. There are discrepancies among publications about what falls under the umbrella of vaccine hesitancy.[12], [13] What vaccine hesitancy is at the population level is hard to characterize because hesitancy is not directly related to vaccine uptake— vaccine-hesitant persons may accept recommended vaccines in a timely manner, despite having doubts in doing so. Furthermore, hesitancy can vary according to the vaccines involved. Vaccine-hesitant individuals are thus a heterogeneous population with varying levels of indecision about some vaccines or about vaccination in general.

By the quantitative vaccination coverage standard, irrespective of the underlying explanations of the phenomenon, French Guiana definitely represents an outlier on the South American continent. Health authorities and Health professionals have continued relentless communication efforts to gain additional percentage points of vaccine coverage but vaccine centers’ activity remains low. In the ongoing argumentative “trench war”[14], reframing the issue of COVID-19 vaccination beyond *pro* and *con* teams could help move forward and restore some everyday life normalcy in French Guiana. Hence, health professionals’ dialog with their patients often successfully counter fake news, reframe the issue and convince them to get vaccinated. In addition, many health professionals try to convince the population by participating in community-based, regional and national radio and television interventions, and by organizing information meetings with the population, community leaders and/or religious leaders, sometimes directly in the remotest villages. At the same time, health mediators from different communities and ethnic groups traveled throughout the territory to explain the issues of the Covid-19 vaccination. [15], [16] News and public health messages mostly report to the public in terms of relative risks and proportion vaccinated metrics that may not be immediately intelligible for many while number of deaths and numbers saved may be easier to grasp. However, so far, to our knowledge, few publications have done so in specific groups of patients but not at the level of the total population.[17], [18], [19] In this perspective, we aimed to be more concrete and reframe the debate in how many deaths and hospitalizations, and costs, the vaccines had and could have avoided, hypothesizing that this may convince undecided opinion leaders of the importance of vaccines and, ultimately, nudge persons to go and get vaccinated.

## 2 Materials and Methods

### 2.1 Number needed to vaccinate (NNV) calculation

A collaborative study in the UK estimated the NNV to prevent one death per year with the formula NNV = 1/(Ix365xCF) with I being the daily incidence and CF the case fatality ratio.[17]Others[20], studying influenza and pneumococcal vaccines, have used a slightly different formula for the NNV to prevent one death per year = 1/(annual population mortality rate attributed to disease in the unvaccinated × vaccine efficacy (VE) for prevention of death due to disease) or 1/(annual attack rate in the unvaccinated × case fatality rate (CF) × vaccine efficacy (VE) for prevention of death due to disease).

We calculated the NNV to prevent one death per year given the incidence numbers of the past 6 months. [17], [20] In contrast with [17]–NNV = 1/(Ix365xCF) where I = daily Incidence, CF = case fatality— we computed the NNV taking into account vaccine efficacy against death NNV = 1/(Ix365xCFxVE). We also calculated the NNV to prevent one death per 6 months given the incidence numbers of the past 6 months, and divided the number of persons vaccinated to estimate how many deaths had been avoided in French Guiana at that time given the mean incidence observed.

We then computed NNV to prevent one death per year for different age groups using age-stratified infection fatality rates and different mean incidence levels. We also computed 95 % confidence intervals based on the uncertainty intervals around the vaccine efficacy.

We also calculated the NNV to prevent one hospitalization and 1 ICU admission per 6 months given the incidence numbers of the past 6 months. We computed the NNV using the BNT162b2 vaccine assuming a 90 % vaccine efficacy (VE) against hospitalization and ICU admission NNV = 1/(Incidencex182.5x(Hr or ICUr)xVE) where Hr is the hospitalization rate or the number of hospitalized cases / number of diagnosed COVID-19 cases and ICUr is the ICU admission rate or the number of admission in ICU for COVID-19 /number of diagnosed COVID-19 cases. [21].

### 2.2 Values used for computations.

Using Santé Publique France epidemiologic surveillance data between April 2021 and October 2021 the mean COVID-19 incidence rate for French Guiana was 406 per million inhabitants. Santé Publique France, a government agency, reports lab-confirmed cases using PCR on nasopharyngeal swabs.[22] Infection fatality rates (IFR) were obtained from [23]. For age group (45–54) the IFR used was 0.23 %, for age group (55–64 years) it was 0.75 %; for age group (65–74 years) it was 2.5 %; for age group (75–84 years) it was 8.5 %; and for age group 85 + years the IFR was 28.3 %.

Santé Publique France provided us epidemiologic surveillance data between April 2021 and October 2021 to estimate the Hr (0.07) and ICUr (0.018). Santé Publique France reports hospitalizations, ICU, and deaths *with* COVID-19 (not necessarily *because* of COVID-19) each week in a bulletin. Costs used for computations were obtained from the Département d’Information Médicale at Cayenne Hospital (see supplementary table for cost computations).

We estimated that the vaccine efficacy (VE) against death and severe disease from the Delta variant of COVID-19 –the currently dominant variant in French Guiana— to be 90 %(95 % CI = 84 to 94).[23] We conducted sensitivity analyses using hypothetical vaccine efficacy rates of 75 %, 80 %, and 85 %.

Vaccination data was obtained from the French health insurance website for French Guiana[4] and from ourworldindata’s website for South America.[24] The overlayed percentage of the population vaccinated was plotted for South American territories.

Finally, we used national estimates of life expectancy per sex by year to compare France and French Guiana.[25].

### 2.3 Ethical and regulatory aspects

The use of aggregated data does not require any particular ethical or regulatory clearance.

## 3 Results

### 3.1 Vaccination numbers and proportions

**Fig. 1:**
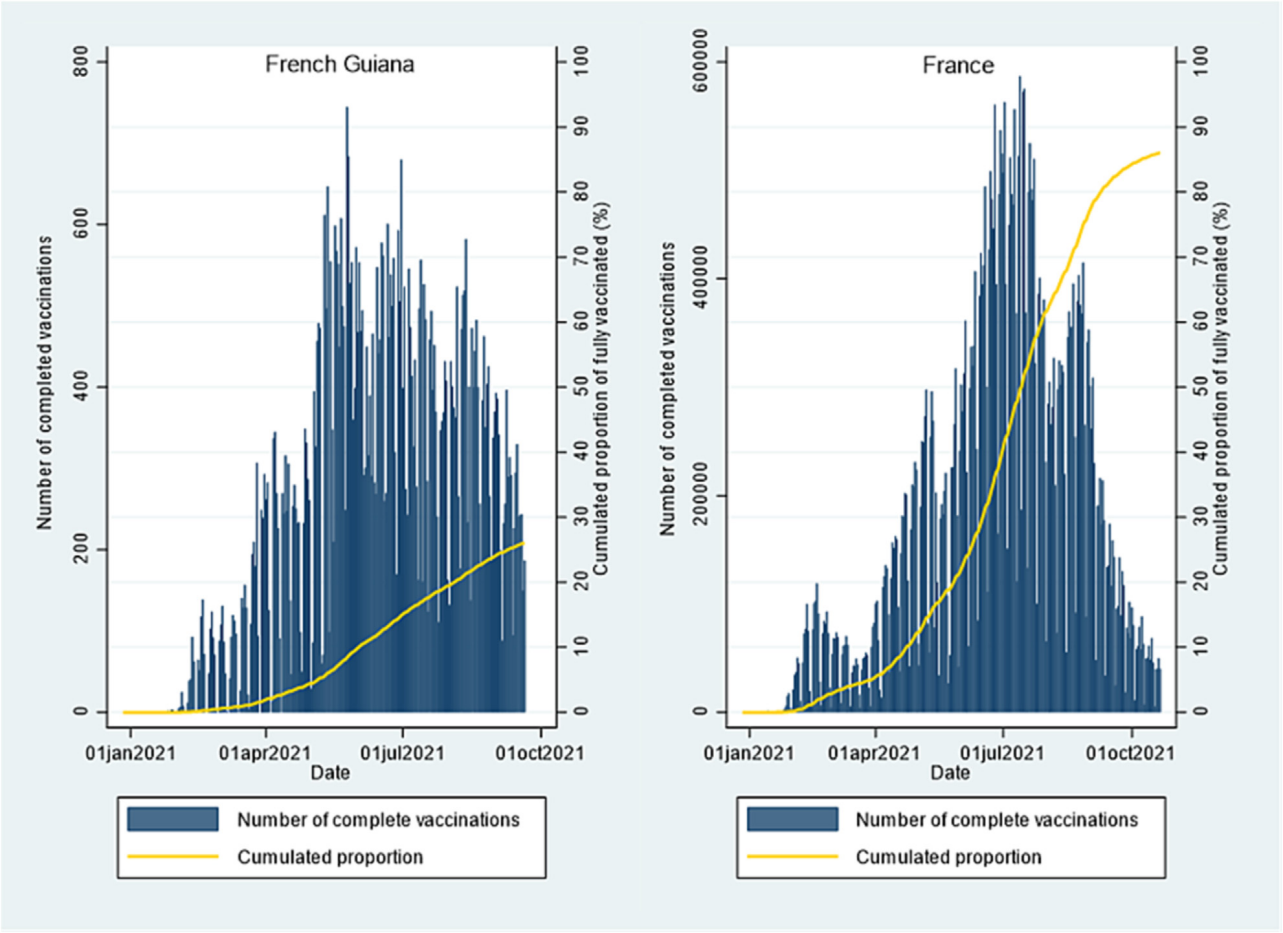
Daily numbers and cumulated proportion of persons vaccinated against COVID-19 for French Guiana and mainland France.

**Supplementary figure 1:**
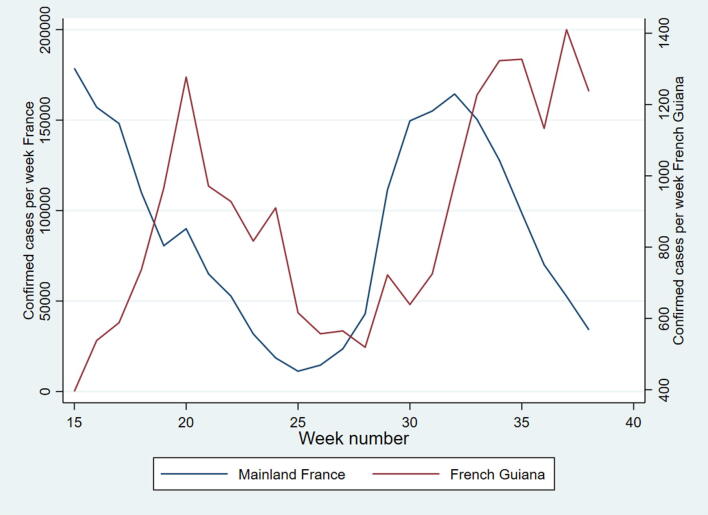


Fig. 1 shows the daily number of completed vaccinations and the cumulated proportion of the vaccinated population. While in mainland France there has been a sharp decline in vaccinations which corresponded to a high proportion of vaccinated and an epidemic drop, in French Guiana the decline has been less marked in a context of sustained transmission and hospital saturation. The cumulated proportion of vaccinated remains low and does not have the sigmoid shape observed in mainland France. In mainland France, the cumulative proportion of vaccinated persons shows that inflexion points align with the diffusion of innovation theory: 16 % *early adopter*, 34 % *early majority*, 34 % *late majority*, and 16 % *laggards*; this was not the case for French Guiana’s cumulated proportion of vaccinated. Supplementary Fig. 1 shows the weekly epidemic curves during the study period for France and French Guiana.

**Fig. 2:**
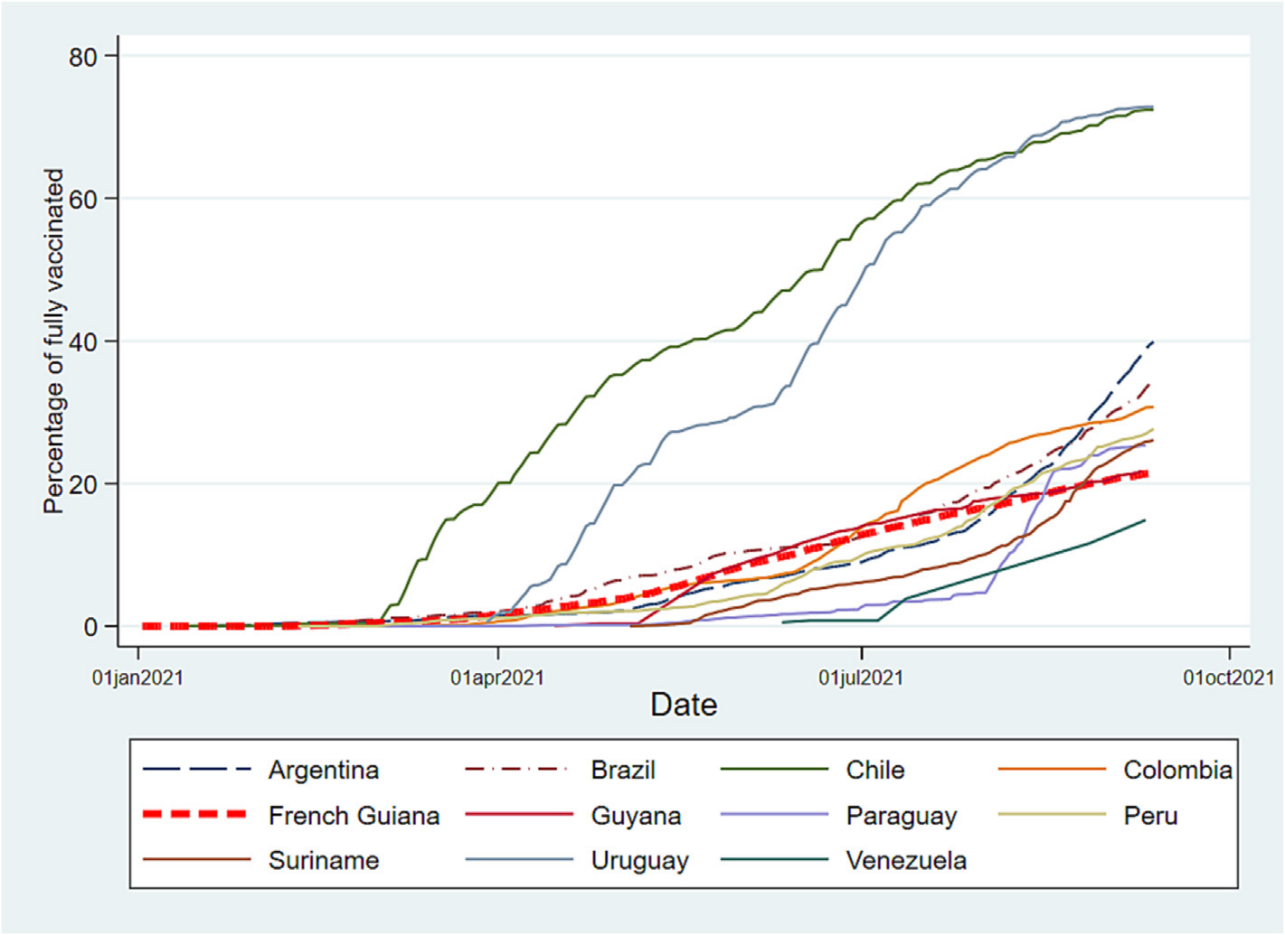
Proportion of the population vaccinated against COVID-19 by date per South American Country or Territory.

Fig. 2 shows the proportion of the population that is fully vaccinated in South American territories. French Guiana started early but the slope has remained weak whereas most other countries have shown periods of marked acceleration.

### 3.2 Crude measures and deaths averted.

The crude number needed to vaccinate to prevent one death per year is 1522 (95 % confidence interval 1457–1631).

In early October, at the end of a 6-month period with an incidence exceeding 400 per million inhabitants, and 148 deaths –most of which were unvaccinated—, French Guiana had vaccinated 71,000 persons, which represents 46 times the NNV to prevent one COVID-19 death per year. Hence, we can crudely estimate that vaccination avoided 46 deaths (IC95%=43.5–48.7). If the number of vaccinated persons reached the same proportion as mainland France (86 % according to the health ministry [3]), this would imply 215,121 persons would be vaccinated hence avoiding 141 deaths per year (IC95%=131.9–147.6). Hence, in the 6 month-long back-to-back Delta and Gamma waves, 61 % (141/232) of deaths would have been avoided. Following this reasoning, the vaccination gap in French Guiana can be estimated to have led to 141–46 = 95 avoidable deaths (95 %CI = 87.5–98.9).

Sensitivity analyses with hypothetical lower vaccine efficacy levels of 75 %, 80 %, and 85 % showed similar results; hence, for such values, vaccination would still have avoided 35, 38, or 40 deaths, respectively; similarly, for the same lower vaccine efficacy hypotheses, if the proportion of vaccinated persons in French Guiana had equaled the proportion in France, 118, 126, or 134 deaths would have been avoided, respectively.

### 3.3 Age and incidence-stratified estimations

**Fig. 3:**
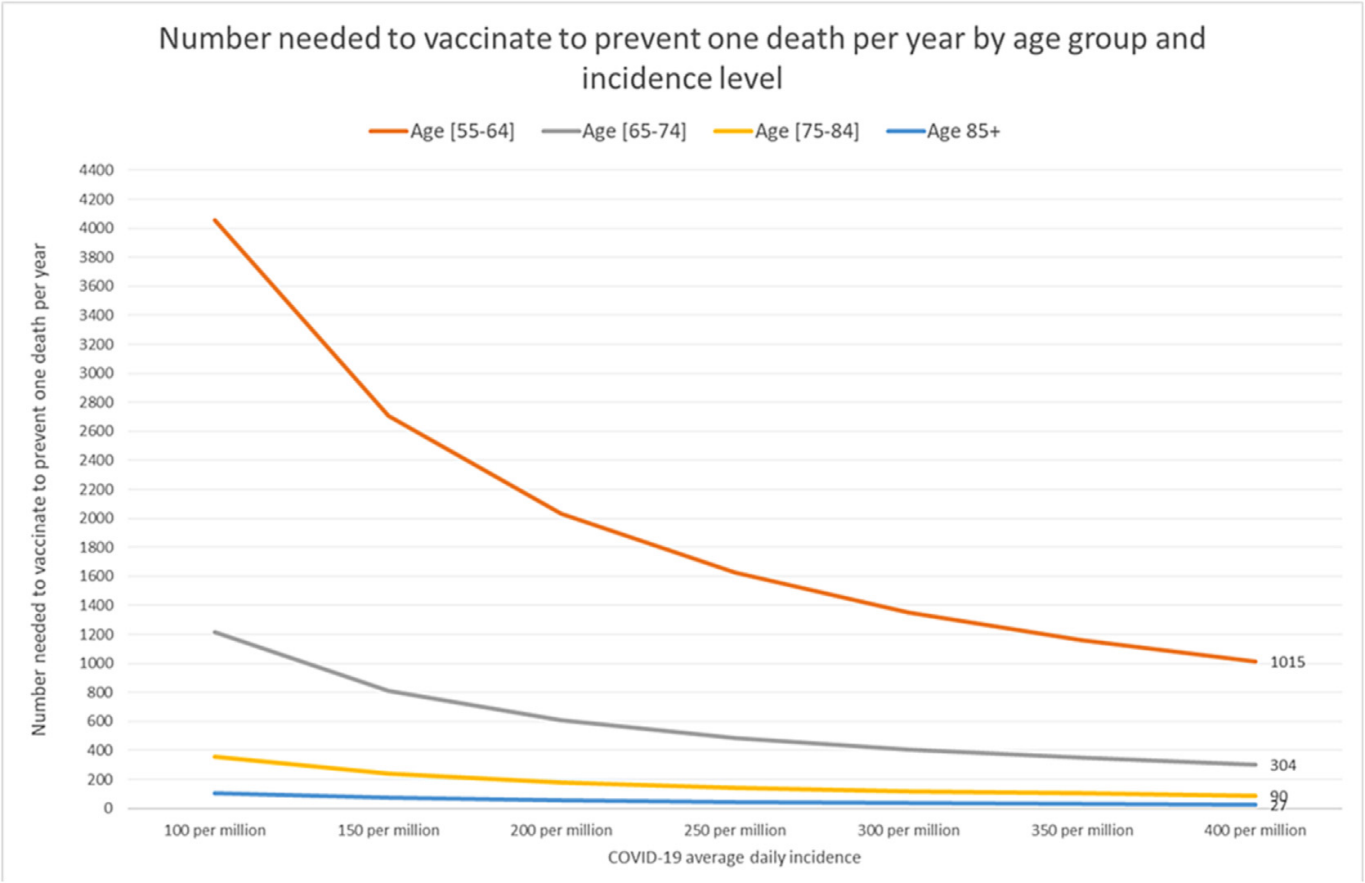
Number needed to vaccinate to prevent one death per year by age group and incidence level.

Fig. 3 shows the estimated NNV for different age groups (starting at 55 years for readability) and epidemic situations (detailed formulas used in supplementary document 1). For the past 6 months, the 400 per million incidence scenario has been the ongoing one showing that for those aged 45 to 54 years 3309 persons vaccinated (95 % CI = 3169–3544) avoided one death; for those aged 55 to 64 years 1015 persons vaccinated (95 % CI = 972–1087) avoided one death; for those 65–74 years 304 persons vaccinated (95 % CI = 291–326) avoided one death; for those aged 75 to 84 years 90 persons vaccinated (95 % CI = 86–96) avoided one death; and that for those aged 85 years or more 27 persons vaccinated (95 % CI = 26–29) avoided one death. The other incidence levels are useful benchmarks for projecting this logic through the different phases of the epidemic and the future evolution of the incidence (250/ million in late October).

**Fig. 4:**
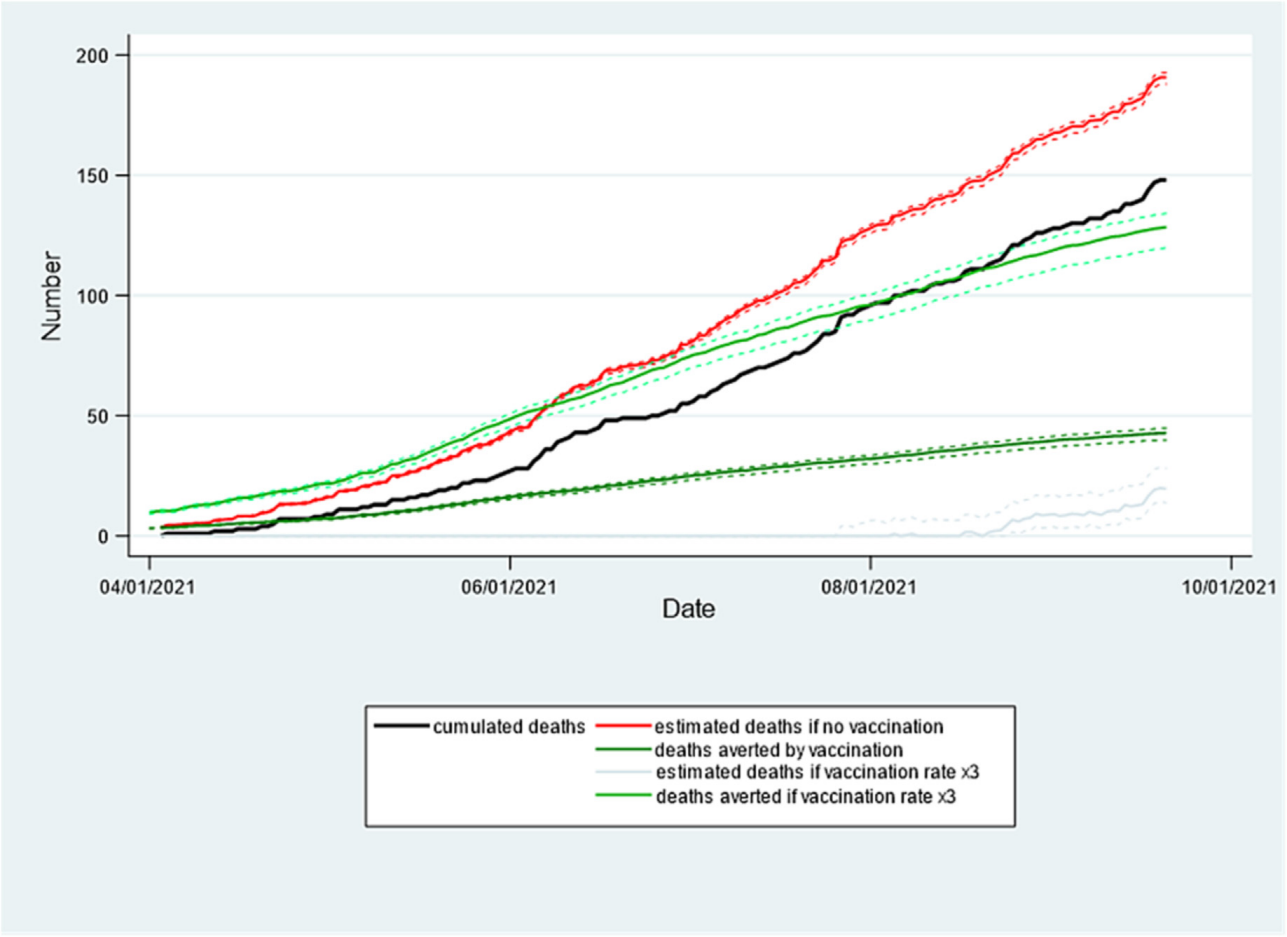
Deaths averted by vaccination in French Guiana at the actual rate and the rate in mainland France. (dashed lines indicate 95% confidence intervals).

Fig. 4 shows the cumulated number of deaths in the study period and the deaths averted by the actual vaccination programme; the figure also estimates the number of averted deaths if vaccination rates had been triple than what they are—as in mainland France; finally, the number of deaths if no vaccines had been available are plotted and the number of deaths if French Guiana had a similar vaccine uptake than mainland France. If French Guiana had not vaccinated anyone, we estimate that the number of deaths since April 2021 would have approached 191; By contrast, if vaccination had reached levels similar to mainland France the cumulated number of deaths would have been 62.

Finally, the number of deaths caused by the vaccine was zero, an important negative result given the intense fear by some of the public.

### 3.4 Hospitalizations, ICU admissions and costs

**Fig. 5:**
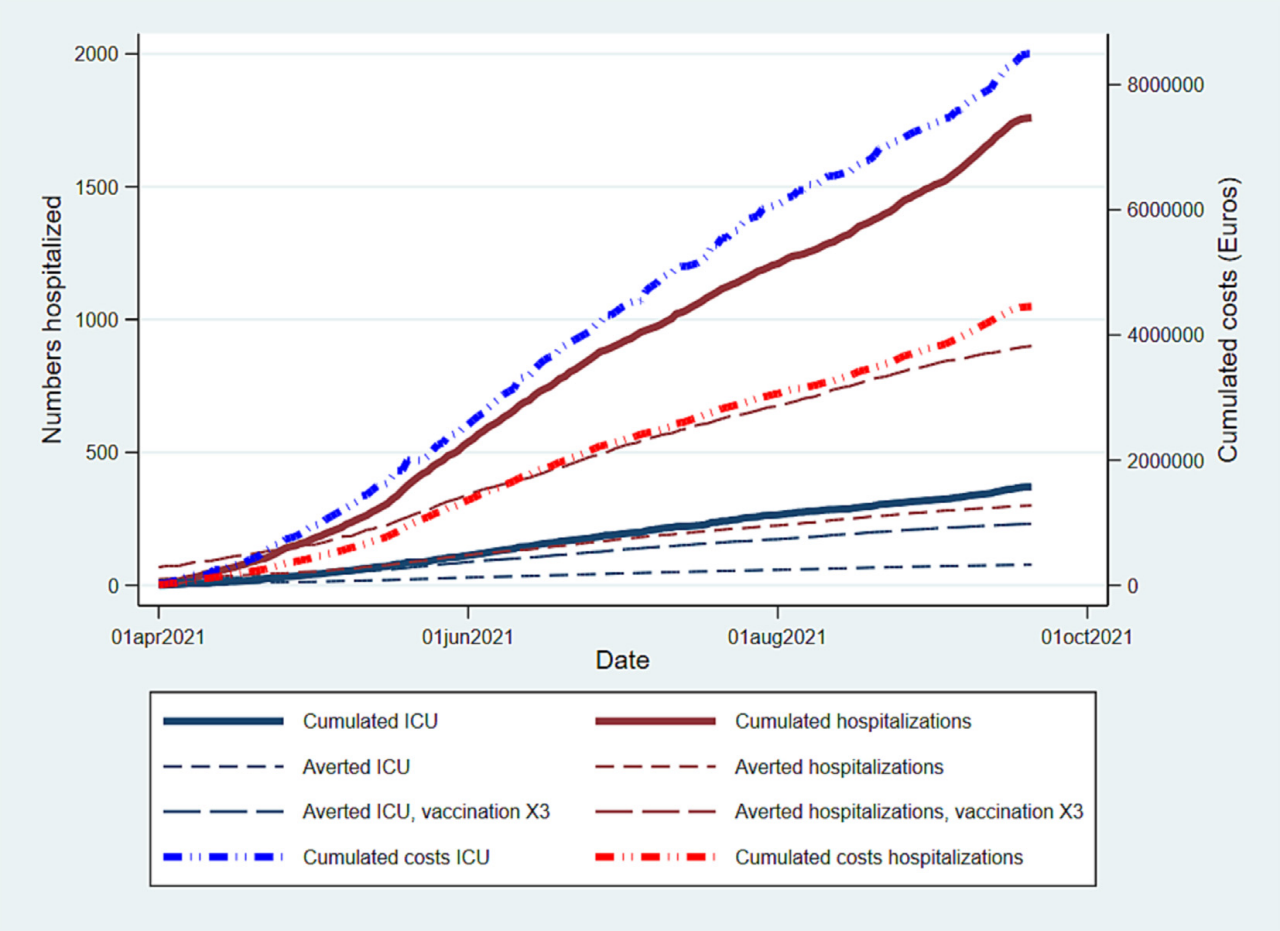
Cumulated hospitalizations and costs, impact of observed vaccination rate and a similar rate than mainland France.

**Fig. 6:**
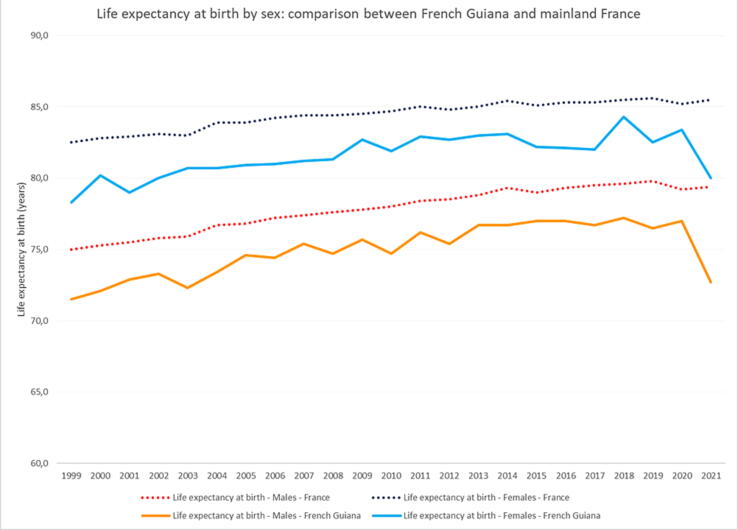
Shows, for males and females, a substantial –5 to 6 years— drop in life expectancy at birth in French.

Based on the observed incidence in French Guiana, hospitalization rate, the crude number needed to vaccinate to prevent one hospitalization per 6 months is 1/(400 per million × 182.5 × 0.07 × 0.9) = 217. Based on our observed incidence and ICU admission rate, the crude number needed to vaccinate to prevent one ICU admission per 6 months is 1/(400 per million × 182.5 × 0.018 × 0.9) = 845. In early October, following a 6-month period with an average incidence exceeding 400 per million inhabitants, with 2085 hospitalization and 370 ICU admissions (total costs = 8,495,200 Euros + 5,280,471 Euros, respectively (cost computations shown in Supplementary Table 1), we estimate that the current albeit low vaccination rate avoided 300 hospital (IC95%=280–313) (724,200 Euros) and 77 ICU admissions (IC95%=72–81) (1,770,338 Euros). (Fig. 5) If the proportion of vaccinated persons had been that of mainland France, we estimate that 900 (2,172,601 Euros) hospitalizations and 231 (5,311,015 Euros) ICU admissions would have been avoided.Fig. 6..

Overall, the vaccine gap relative to mainland France means that there would have been “only” 1185 hospitalizations and that there was an excess of 43 % of hospitalizations burdening the hospital system and afflicting patients and families.

**Supplementary figure 2:**
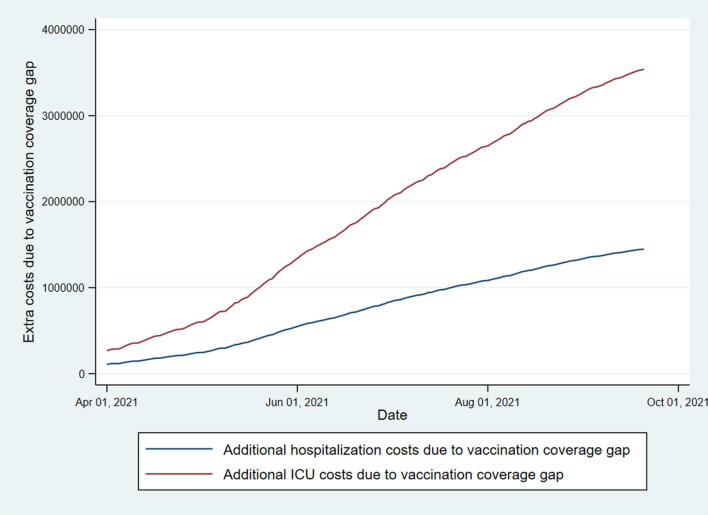


**Supplementary figure 3:**
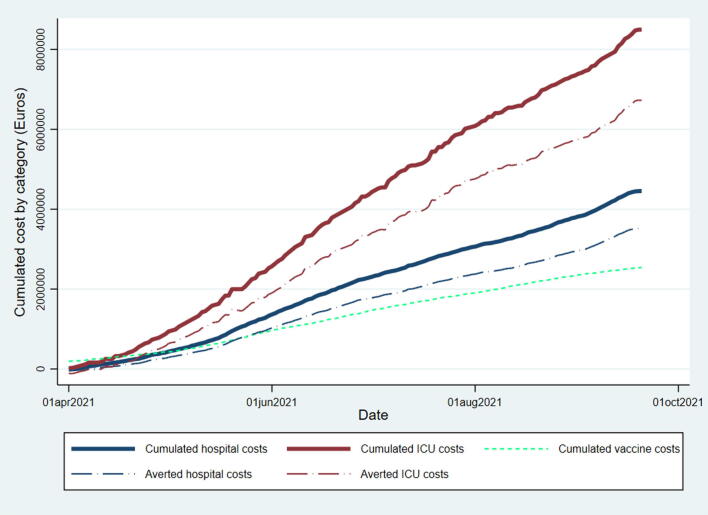


The vaccine gap relative to mainland France means that if the dynamics of vaccine uptake had been similar to mainland France, there would have been “only” 139 ICU admission (instead of 370) and that 62.4 % of ICU stay burdening the hospital system and afflicting patients and families could have been avoided. Supplementary Figs. 2 and 3 respectively show the cost of the vaccination gap through the studied period and detailed costs and averted costs through the period.

The low vaccine uptake, despite the costs of vaccination, nevertheless averted numerous hospitalizations and costs (Supplementary Fig. 3).

### 3.5 Evolution of life expectancy at birth in French Guiana and mainland France

Guiana relative to mainland France, which was also hit hard by COVID-19 but had greater vaccine coverage.

## 4 Discussion

The present study estimates that 46 deaths were prevented by the vaccine in French Guiana during a 6-month period covering back-to-back COVID-19 waves; The study also estimates that, in this French territory, if coverage had progressed similarly to mainland France, 141 (61 %) of the fatalities would have been avoided. The vaccine coverage gap between France and French Guiana led to 95 additional deaths –a substantial number in a sparsely populated territory which counted a total of 970 deaths in 2017, before the epidemic.[26] Here we also show that despite low vaccination rates, the COVID-19 vaccine avoided a substantial number of ICU and classical hospitalizations, hence perhaps allowing the hospital system to resist in the face of the back-to-back third and fourth waves. However, the numbers computed for the vaccination gap—if French Guiana had the same vaccination dynamic as mainland France— show that much of the suffering and straining of the hospital system could have been avoided if the French Guianese society had not resisted vaccination. This was reflected in the substantial drop in life expectancy at birth in 2021 in French Guiana relative to mainland France.

The costs measurements here are incomplete: the costs of flying in medical and paramedical reinforcements throughout the crisis to open addition ICU beds and relieve exhausted health professionals were not accounted for; the costs of postponing most surgical procedures, of interrupting follow-up of chronic diseases, of testing and care of out of hospital patients, contact tracing costs, the lack of health insurance income due to COVID-19 related unemployment were also not accounted for. On the vaccination side, the costs of setting up vaccine centers and staffing them are not included in this tally. Overall, the excess costs due to COVID-19 hospitalization are likely to be even greater than what we have estimated. The imperfect above calculations nevertheless give an intelligible order of magnitude at the scale of our territory. The total numbers and costs may seem small compared to other countries but for French Guiana and its fragile hospital system, they are quite substantial.

It remains to be seen whether the more specific perspective of “what did not happen” because of vaccination will have the cognitive sway to convince unvaccinated people to get vaccinated. However, the numbers of what should not have happened show the tragic consequences of vaccine hesitancy or resistance, and misinformation. Framing the problem, taking cognitive biases into account when communicating may substantially impact behavior.[27], [28] In this sense, we hope that the numbers produced here may help reframe the issue of vaccination more meaningfully in a population that underplays or even denies the risk, as if French Guiana, because of its youth and natural remedies, was immune to this global problem. However, this complacency about the disease overlooks the high prevalence of hypertension, obesity and diabetes in French Guiana, 3 risk factors for severe COVID-19 that are more prevalent than in mainland France[29].

Research in the diffusion of innovations[30] theorize that in societies the population can be broken down into 4 (or 5) groups according to their willingness to accept an innovation with 16 % of so called “early adopters”, 34 % of the “early majority”, 34 % of the “late majority”, and 16 % of “laggards”. Using the diffusion of innovation framework[31], the comparison of the vaccine uptake curves in mainland France and French Guiana (Fig. 1) suggests that while in France the cumulative curve is fairly in agreement with the theory –—French Guiana’s cumulative distribution does not follow the same sigmoid shape, emphasizing the acuteness and the complexity of the problem[32]. The phased roll-out, gradually expanding eligibility, is likely to explain some of the jaggedness in the distribution, but overall the inflexion point in the curve fit nicely with the theory in mainland France; by contrast in French Guiana, where opposition forces have been far greater, the aspect seems more aligned with the concept of failed diffusion[33]. Vaccine availability bottlenecks have never been an issue in French Guiana –outreach vaccination programmes were deployed even in the most remote villages in the Amazonian forest— therefore the curve reflects the rate of adoption of the innovation. In this culturally and ethnically diverse society, social network theory suggests failed diffusion may reflect the overconnection of some networks reflecting the balance between homophily and heterophily.[34], [35] This failed diffusion in the populational network may also reflect the lack of local involvement and community participation; it notably questions the role of French Guiana’s opinion leaders who have the most influence in evaluating and influencing the decision process of late adopters.[36] There is some hope, as in September, a number of opinion leaders have started to promote vaccination.[11] In a population that has long underplayed or even denied the risk of COVID-19, the concrete consequences of vaccine hesitancy or resistance, and misinformation could help local community opinion leaders and elected officials express the consequences of this vaccine gap and enhance their leadership potential in a complex heterogenous society.

The total numbers may seem small compared to large countries like the USA or Brazil, but at the small scale of French Guiana, they are a big deal: The fact that, in 2021, part of a rich territory has lost nearly 100 persons because of vaccine hesitancy and insufficient implication of opinion leaders is hard to ignore. The history of AIDS in Africa has shown that leadership among opinion leaders –and among them elected politicians— is crucial to navigate health crises and accelerate behavior change.[37], [38], [39], [40] As discussed above the social networks’ properties are presumably also at play in hampering the diffusion of acceptance of vaccination.

The French law of modernization of the health system, has emphasized health promotion at all ages and in all policies, illustrating a view where all sectors of society –not just health professionals—contribute to health. The recent emphasis on health democracy, also very present in French Guiana, is embodied by the regional commission for autonomy and health, which so far has produced no concrete recommendations despite the acuteness of the situation. Furthermore, the sobering fact is that, in the present crisis, the most successful gains came not so much from enlightened and empowered citizens responsibly optimizing their health, but from government-imposed curfews, masks mandates, and medical innovation and strong incitements to get vaccinated.

The limitations of the crude calculations presented here are notably that the population of French Guiana is young and that we did not have the breakdown of hospitalizations or deaths by age. The official data compiled hospitalizations, ICU admissions or deaths with COVID which may not always mean caused by COVID and cause an overestimation of actual numbers. Nevertheless, they give an intelligible order of magnitude at the scale of our territory. The confidence intervals of vaccine efficacy, of incidence levels, and the precise prevalence of comorbidities may have added further uncertainty to the estimations. Sensitivity analyses using vaccine efficacy levels of 75 %, 80 %, or 85 % still showed substantial differences between French Guiana and mainland France. Another limitation, which may have led to underestimate the burden of COVID-19, is that crude incidence and case fatality rates used in the computations are actually different between vaccinated and unvaccinated persons—they are greatest in the unvaccinated. Thus the number needed to vaccinate to prevent 1 adverse outcome is likely to be overestimated whereas the number of averted deaths is underestimated, as are the estimations of avoidable deaths if vaccination coverage had equaled that achieved in mainland France.

Despite these study limitations, producing such estimates for a given territory may help future health communication by providing numbers that are easier to grasp for a public that has been exposed to daily numbers of COVID-19 deaths. Although there may be distrust towards government sources, for trusted sources such as family physicians, pharmacists or nurses interacting with hesitant patients, the present results provide concrete and striking arguments that may be useful to convince some patients to accept to get vaccinated.

From the perspective of South America, apart from French Guiana, the 3 territories with the lowest initial vaccine protocol coverage are Suriname (40 %), Guyana (46 %) and Bolivia (49 %).[24] Although Bolivia, Suriname and Guyana are among the poorest countries in terms of GDP per capita[41], French Guiana is not. Another feature of these countries is that they are among the most ethnically diverse and have the lowest proportion of European ancestry.[42] In view of these characteristics, the low vaccine uptake in these territories perhaps reflects insufficient social cohesion where distrust and disconnection fall along ethnic and socioeconomic lines.

How to break entrenched positions is still a conundrum far beyond French Guiana. In addition to elements pertaining to network properties, some authors have used a social marketing lens to understand and improve vaccine coverage.[43] In this perspective creating vaccine demand focuses on key elements, including vaccine branding, service marketing in relation to vaccine distribution, supply-side trust, and competitive strategy. [43] A recent systematic review evaluated nudging interventions.[44] These include changing the way information is framed and delivered to a target audience, changing the messengers who provide the information, invoking social norms and emotions (e.g., through storytelling, dramatized narratives, and graphic presentations), using reminders, offering incentives or changing default options. The effectiveness of nudging interventions and the direction of effect vary considerably by context. The results are sometimes mixed, underscoring the need for further research, notably on the transferability of effective interventions to different contexts.[44].

## 5 Conclusions

In conclusion, there is a nearly 3-fold difference in the proportion of vaccinated against COVID-19 between mainland France and French Guiana, a coverage gap that has led to numerous avoidable deaths, hospitalizations, and costs. The coverage gap has also led to overwhelm the hospital system and exhaust health professionals. Although an estimated 46 deaths were prevented by the COVID-19 vaccine in French Guiana during the study period; the study also estimates that if coverage had been similar to mainland France, by contrast 141 (61 %) of the fatalities would have been avoided. Overall, the vaccine gap relative to mainland France means that there would have been “only” 1185 hospitalizations and that there was an excess of 43 % of hospitalizations burdening the hospital system and afflicting patients and families. Furthermore, if the dynamics of vaccine uptake had been similar to mainland France, there would have been “only” 139 ICU admission (instead of 370) and that 62.4 % of ICU stay burdening the hospital system and afflicting patients and families could have been avoided. In a territory with a total population of circa 300 000 persons, these numbers are substantial and their communication may help in improving vaccine coverage in French Guiana, the South American territory with, by far, the greatest health expenditure per capita but also the lowest vaccination coverage.

## Funding

“This research received no external funding”.

## Institutional Review Board Statement

“Not applicable.”

## Data Availability Statement

The data is already publicly available at data.gouv.fr and ourworldindata.org.

## CRediT authorship contribution statement

**Mathieu Nacher:** Conceptualization, Methodology, Formal analysis, Writing – original draft, Writing – review & editing. **Nicolas Vignier:** Validation, Writing – review & editing. **Cyril Rousseau:** Validation, Resources, Data curation, Writing – review & editing. **Antoine Adenis:** Validation, Writing – review & editing. **Maylis Douine:** Writing – review & editing. **Célia Basurko:** Writing – review & editing. **Bertrand de Toffol:** Validation, Writing – review & editing. **Narcisse Elenga:** Validation, Writing – review & editing. **Hatem Kallel:** Validation, Writing – review & editing. **Jean Pujot:** Validation, Writing – review & editing. **Magaly Zappa:** Validation, Writing – review & editing. **Magalie Demar:** Validation, Writing – review & editing. **Félix Djossou:** Validation, Writing – review & editing. **Pierre Couppié:** Validation, Writing – review & editing. **Loïc Epelboin:** Validation, Writing – review & editing.

## Declaration of Competing Interest

The authors declare that they have no known competing financial interests or personal relationships that could have appeared to influence the work reported in this paper.

## Data Availability

Data will be made available on request.
