# Supplementary material for: The burden of COVID-19 in French Guiana: Vaccine-averted deaths, hospitalizations and costs
Source: Vaccine X. 2023 Feb 11;13:100271. doi: 10.1016/j.jvacx.2023.100271 (PMC9918439; doi:10.1016/j.jvacx.2023.100271)
Supplement: Supplementary data 4 [file mmc4.docx]

| **Item** | **Cost per unit** | **Mean duration of stay** | **Mean total cost per patient in Euros (durationxcost per day)** | **Number of patients** |
| --- | --- | --- | --- | --- |
| **Hospitalization** | 603 Euros per day | 4 | 2,412 | 2,412xN |
| **Intensive Care Unit** | 1025 Euros per day | 22.4 | 22,960 | 22,960xN |
| **Vaccine** | 19.5 euros per dose | 2* | 39 | 39xN |

Supplementary table. **Table of cost calculations.**

**2 dose protocol at the time*
